# Introduction to laser thermal therapy for brain disorders

**DOI:** 10.1080/02656736.2020.1789764

**Published:** 2020-07

**Authors:** Jennifer S. Yu, Alireza M. Mohammadi

**Affiliations:** a Department of Radiation Oncology, Taussig Cancer Institute, Cleveland Clinic, Cleveland, OH, USA; b Department of Cancer Biology, Lerner Research Institute, Cleveland Clinic, Cleveland, OH, USA; c Department of Neurosurgery, Neurological Institute, Cleveland Clinic, Cleveland, OH, USA

Hyperthermia has long been recognized in medicine for its therapeutic properties. While hyperthermia has become widely used extracranially for many years, its use in the brain has been hampered by technical and safety concerns. A phase-III clinical trial with microwave hyperthermia led to prolongation of survival in patients with glioblastoma, a lethal primary brain tumor [1]. Yet, technical challenges in microwave interstitial catheter implantation, limitations in thermometry, quality and safety concerns, and lack of programs to train physicians and surgeons in this technique led to its abandonment. Unfortunately, the early success with brain hyperthermia was cast aside. Mainstream medicine moved on to new chemotherapies, targeted agents and immunotherapy.

The last few years have ushered a resurgence of hyperthermia applications in the brain. Advances in laser, ultrasound and MRI technologies have improved the delivery and quality of hyperthermia. Stereotactic mapping of targets coupled with real-time thermometry has enabled safe delivery of hyperthermia. These advances have renewed interest in hyperthermia in the management of brain tumors. Additionally, indications have expanded to include treatment of radiation necrosis, a complication of radiosurgery commonly used to treat brain metastases, and functional disorders including epilepsy. Due to its minimally invasive technique, brain hyperthermia, particularly laser ablation, offers an appealing alternative to traditional craniotomy. With reduced recovery time and length of hospitalization, patients may go on to radiation therapy or systemic treatment more quickly. The clinical and economic value of brain hyperthermia is increasingly being realized.

Moreover, in this era of immunotherapy, hyperthermia may be regarded as a tool for creating personalized, *in situ* cancer vaccines (readers are referred to the special issue on Thermal Therapy and Immunotherapy: at the Crossroads of New Discovery [2]). Hyperthermia has numerous effects on cancer cells and their surrounding microenvironment. Direct tumoricidal effects of ablative hyperthermia and thermal effects within lower temperature zones sensitize cancer cells, including cancer stem cells, to radiation and chemotherapy [3,4]. Consequently, tumor antigen spillage can provoke an antitumor immune response. The lower temperature penumbra has numerous effects including increasing blood perfusion, improving tissue oxygenation to render radiation more effective, improving chemotherapy penetration into the tumor, and augmenting immune cell recruitment [5]. Emerging data also support the role of hyperthermia in disrupting the blood-brain-barrier and/or blood-tumor-barrier [6]. These insights inform how hyperthermia can be integrated with other treatment modalities including immunotherapy to take full advantage of the biologic consequences of hyperthermia.

In this special issue, leading experts share new advances in the biology and clinical delivery of brain hyperthermia. Skandalakis [7] provide an overview of hyperthermia approaches in the management of brain tumors including laser ablation, magnetic nanoparticle and photothermal therapies. Frenster [8] discuss modes of cancer cell death induced by hyperthermia and their implications for enhancing immune cell surveillance. Srinivasan [9] review advances in thermal therapy in modulating the tumor immune microenvironment and provide strategies for integrating hyperthermia and immunotherapy. Patel [10] assess the effects of laser ablation and ultrasound on the integrity of the blood-brain-barrier and blood-tumor-barrier, and its implications for the delivery of drug and antibody-based therapies to the tumor.

A series of articles assess the role of hyperthermia in the management of malignant and benign diseases. Avecillas-Chasin [11] review advances of laser ablation for the treatment of primary and recurrent gliomas, nuances of hyperthermia delivery and on-going clinical trials. Bastos [12] discuss the role of laser ablation in the management of brain metastases. Dhawan [13] discuss the cost-effectiveness of laser therapy for primary brain tumors and brain metastases. Hong [14] evaluate the role of laser ablation in the treatment of radiation necrosis in the context of brain metastases and propose its use to treat radiation necrosis in the setting of benign diseases. Zemmar [15] discuss the role of hyperthermia in adult patients with epilepsy. Finally, Remick [16] discuss emerging indications for the treatment of pediatric patients, with an emphasis on epilepsy stemming from various etiologies.

## References

[1] Sneed PK, Stauffer PR, McDermott MW, Survival benefit of hyperthermia in a prospective randomized trial of brachytherapy boost +/− hyperthermia for glioblastoma multiforme. Int J Radiat Oncol Biol Phys. 1998;40(2):287–295. 10.1016/s0360-3016(97)00731-1 9457811

[2] Fiering SN, Evans S. Introduction to thermal therapy and immunotherapy: at the crossroads of new discovery. Int J Hyperthermia. 2019;36(sup 1):1–2. 10.1080/02656736.2019.1659427 31795831

[3] Man J, Hyperthermia sensitizes glioma stem-like cells to radiation by inhibiting AKT signaling. Cancer Res. 2015;75(8):1760–1769. 10.1158/0008-5472.CAN-14-3621 PMC4401644 25712125

[4] Atkinson RL, Zhang M, Diagaradjane P, Thermal enhancement with optically activated gold nanoshells sensitizes breast cancer stem cells to radiation therapy. Sci Transl Med. 2010;2(55):55ra79. 10.1126/scitranslmed.3001447 PMC4123313 20980696

[5] Chu KF, Dupuy DE. Thermal ablation of tumours: biological mechanisms and advances in therapy. Nat Rev Cancer. 2014;14(3):199–208. 10.1038/nrc3672 24561446

[6] Leuthardt EC, Duan C, Kim MJ, Hyperthermic laser ablation of recurrent glioblastoma leads to temporary disruption of the peritumoral blood brain barrier. PLOS One. 2016;11(2):e0148613. 10.1371/journal.pone.0148613 PMC4766093 26910903

[7] Skandalakis G, Rivera D, Rizea C, Hyperthermia treatment advances for brain tumors. Int J Hyperthermia. 2020;37:3–19. 10.1080/02656736.2020.1772512 PMC7756245 32672123

[8] Frenster J, Desai S, Placantonakis D. In vitro evidence for glioblastoma cell death in temperatures found in the penumbra of laser-ablated tumors. Int J Hyperthermia. 2020;37:20–26. 10.1080/02656736.2020.1774082 PMC7725000 32672127

[9] Srinivasan E, Sankey E, Grabowski M, The intersection between immunotherapy and laser interstitial thermal therapy: a multipronged future of neuro-oncology. Int J Hyperthermia. 2020;37:27–34. 10.1080/02656736.2020.1746413 PMC11229985 32672126

[10] Patel B, Yang P, Kim A. The effect of thermal therapy on the blood-brain barrier and blood-tumor barrier. Int J Hyperthermia. 2020;37:35–43. 10.1080/02656736.2020.1783461 PMC7709344 32672118

[11] Avecillas-Chasin J, Atik A, Mohammadi A, Laser thermal therapy in the management of high-grade gliomas. Int J Hyperthermia. 2020;37:44–52. 10.1080/02656736.2020.1767807 32672121

[12] Bastos D, Fuentes D, Traylor J, The use of laser interstitial thermal therapy in the treatment of brain metastases: a literature review. Int J Hyperthermia. 2020;37:53–60. 10.1080/02656736.2020.1748238 32672122

[13] Dhawan S, Bartek J, Chen C. Cost-effectiveness of stereotactic laser ablation (SLA) for brain tumors. Int J Hyperthermia. 2020;37:61–67. 10.1080/02656736.2020.1774084 32672125

[14] Hong C, Beckta J, Kundishora A. Laser interstitial thermal therapy for treatment of cerebral radiation necrosis. Int J Hyperthermia. 2020;37:68–76. 10.1080/02656736.2020.1760362 32672119

[15] Zemmar A, Nelson B, Neimat J. Laser thermal therapy for epilepsy surgery: current standing and future perspectives. Int J Hyperthermia. 2020;37:77–83. 10.1080/02656736.2020.1788175 32672124

[16] Remick M, McDowell M, Gupta K. Emerging indications for stereotactic laser interstitial thermal therapy in pediatric neurosurgery. Int J Hyperthermia. 2020;37:84–93. 10.1080/02656736.2020.1769868 32672117

